# A multitask Transformer-VAE framework for robust PPG denoising and atrial fibrillation screening

**DOI:** 10.1186/s12880-026-02399-9

**Published:** 2026-05-16

**Authors:** Lingyu Zhang, Lin Yuan

**Affiliations:** 1 School of Physics and Electronic Engineering, Sichuan University of Science and Engineering, Zigong, 643000 China; 2 Key Laboratory of Higher Education of Sichuan Province for Enterprise Informationalization and Internet of Things, Sichuan University of Science and Engineering, Zigong, 643000 China; 3 College of Electronic Engineering, Chengdu University of Information Technology, Chengdu, 610225 China

**Keywords:** Photoplethysmography (PPG), Atrial fibrillation detection, Multitask learning, Variational autoencoder, Transformer model, Feature projection, Signal reconstruction

## Abstract

**Background:**

Photoplethysmography (PPG) is a noninvasive biosignal widely used for atrial fibrillation (AF) screening via wearable devices. However, reliable PPG-based detection remains challenging because motion artifacts, noise contamination, and intersubject variability distort waveform morphology and compromise diagnostic consistency.

**Methods:**

We propose an end-to-end dual-task model that denoises PPG signals and detects AF simultaneously. The model uses a transformer-based encoder with two task branches and an additional lightweight alignment constraint to encourage the two tasks to learn consistent representations under motion artifacts. We trained and evaluated the framework on public PPG datasets, including an internal cohort of 30,773 segments from 91 patients and an external dataset derived from the MIMIC-III waveform database. Performance was evaluated via the AUC and accuracy, together with robustness tests across different noise conditions. We also applied an A-Test procedure that repeats balanced k-fold validation with different k values to assess error stability across data splits.

**Results:**

The proposed model achieved an AUC of 0.9097 and an accuracy of 88.4%, outperforming conventional convolutional and single-task baselines. Robustness experiments demonstrated stable performance across varying signal‒to‒noise ratios. The dual-task architecture preserved physiologically relevant rhythm features, including heart rate variability. A-Test evaluation indicated stable error behavior across data splits, suggesting reduced partition sensitivity and improved learning consistency.

**Conclusions:**

This Transformer–VAE framework integrates signal reconstruction and diagnostic learning to enable accurate and noise-resilient AF detection from PPG signals. Within a healthcare workflow, the model supports wearable screening by generating AF risk indicators and signal-quality cues that can trigger confirmatory single-lead ECG assessment and appropriate clinical follow-up.

**Trial registration:**

Not applicable.

## Highlights

Developed a Transformer–VAE multitask model for joint PPG reconstruction and AF detection.

Introduced feature alignment to enhance representation consistency across dual tasks.

Achieved superior AUC (0.9097) and robustness under severe motion artifacts.

Preserved heart rate variability features, ensuring physiological interpretability.

Provides a scalable framework for reliable AF screening from wearable PPG signals.

## Graphical Abstract

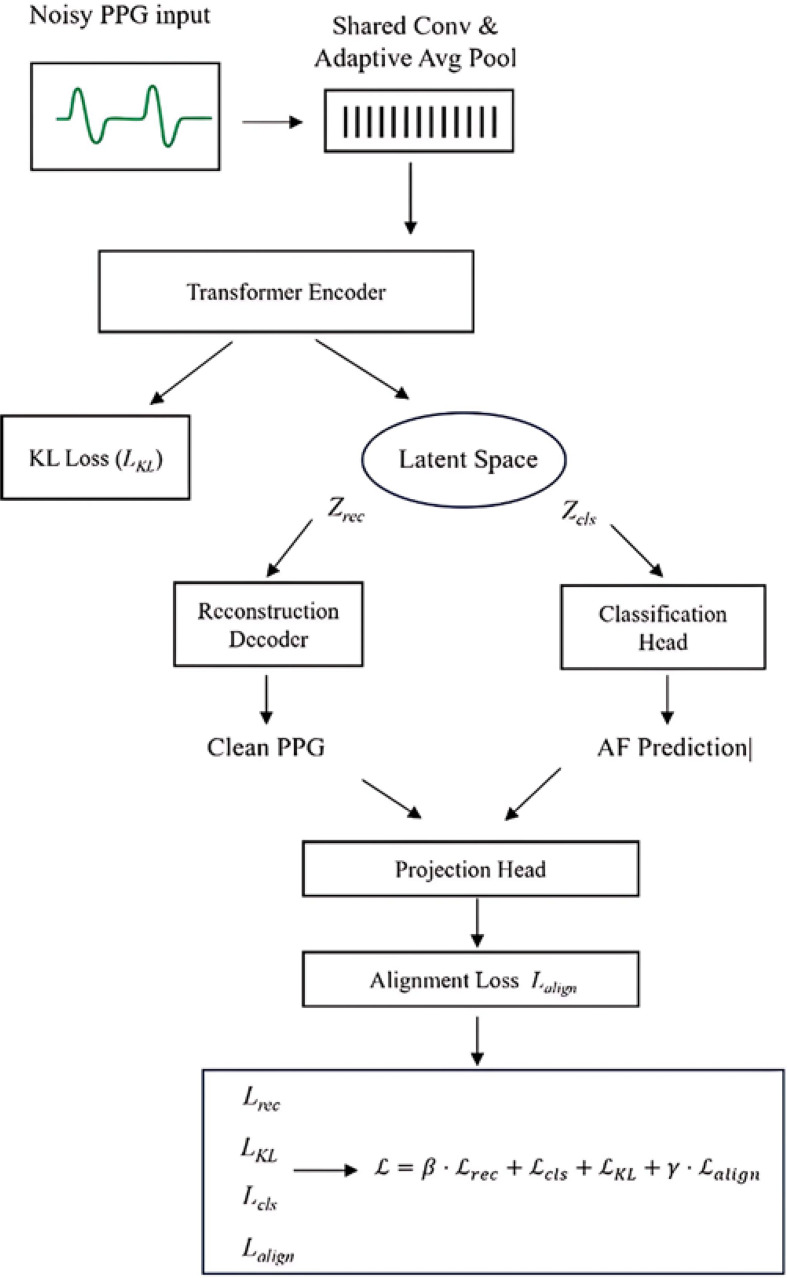

## Introduction

Atrial fibrillation (AF) is the most common type of sustained cardiac arrhythmia, affecting more than 33 million individuals globally, with its prevalence in the United States projected to exceed 12 million by 2050 [1]. As a major contributor to stroke, heart failure, and premature mortality, AF remains challenging to detect because it is paroxysmal and often asymptomatic. Clinically, AF is categorized into several temporal patterns that differ in persistence and management implications. Paroxysmal AF consists of self-terminating episodes that typically last less than seven days and often resolve within 24 h. Persistent AF persists beyond seven days and usually requires medical or electrical intervention for termination. Long-standing persistent AF refers to sustained arrhythmia lasting more than one year, whereas permanent AF denotes cases in which rhythm control is no longer pursued. These varying AF patterns produce heterogeneous rhythm signatures, ranging from intermittent irregularity to sustained beat-to-beat variability. This variability complicates reliable detection, particularly in wearable monitoring environments where the signal quality may fluctuate. Consequently, robust signal modeling is essential for distinguishing pathological rhythm irregularities from motion-induced artifacts.

Electrocardiogram (ECG) remains the clinical gold standard for AF detection due to its direct measurement of cardiac electrical activity. Conventional ECG-based screening, while clinically accurate, is constrained by short monitoring windows and the need for specialized equipment, underscoring the demand for long-term, unobtrusive monitoring approaches.

Photoplethysmography (PPG) offers a low-cost and noninvasive alternative for tracking peripheral blood volume changes. Its integration into consumer wearable devices such as smartwatches and fitness bands enables continuous, large-scale cardiovascular monitoring beyond clinical settings [2]. Importantly, single-lead ECG sensors embedded in modern wearable devices already provide clinically meaningful AF screening capabilities and are generally less susceptible to motion artifacts than optical PPG measurements. Therefore, PPG should not be viewed as a replacement for ECG-based diagnosis but rather as a complementary modality that enables continuous, passive physiological monitoring where optical sensing is more practical. Compared with traditional multilead clinical ECG systems, PPG sensors offer advantages in wearability, scalability, and user comfort, making them attractive for long-term population monitoring when signal robustness can be ensured [3].

The rapid advancement of deep learning has significantly improved the ability to extract meaningful physiological information from PPG signals. Recent studies employing convolutional neural networks (CNNs), recurrent neural networks (RNNs), and transformer architectures have achieved competitive results in tasks such as blood pressure estimation, arrhythmia detection, and signal denoising [4–6]. For example, the PPGnet model performed well on the EMBC 2022 dataset for blood pressure prediction [7, 8], and CNN-LSTM hybrid models have shown robustness under noisy conditions in continuous BP estimation [9–11].

Despite this progress, PPG-based AF detection still faces several persistent challenges. First, motion artifacts and sensor displacement frequently introduce waveform distortion and low signal-to-noise ratios (SNRs), particularly in ambulatory environments [12, 13]. These artifacts disproportionately affect optical sensing compared with electrical ECG measurements, making robust PPG reconstruction a prerequisite for reliable downstream analysis. Second, intersubject variability, arising from skin tone, vascular elasticity, or contact pressure, limits model generalizability [14–16]. Third, severe class imbalance, where normal rhythm segments overwhelmingly outnumber AF samples, reduces classifier sensitivity in real-world applications [17, 18].

Previous efforts to mitigate these issues have focused on three paradigms: (1) signal denoising as a preprocessing stage using wavelets or autoencoders [19, 20]; (2) feature enhancement via synthetic signal generation via GANs or VAEs [21, 22]; and (3) joint learning frameworks for reconstruction and classification, often without explicit coordination between tasks [23, 24]. These approaches either treat tasks in isolation or lack mechanisms to regulate task interaction and information flow.

In this work, we propose a multitask learning (MTL) framework that jointly performs PPG signal reconstruction and AF classification, addressing denoising and rhythm discrimination within a unified learning architecture. By integrating a shared encoder with task-specific decoders, the model enables bidirectional supervision between generative and discriminative objectives: the classification branch imposes physiologically meaningful constraints that stabilize the reconstruction, whereas the reconstruction branch enriches latent representations to improve AF discrimination under noisy conditions. Inspired by prior successes of MTL in ECG and EEG analysis [25], the novelty of our approach lies in explicitly coordinating these dual objectives through a feature-alignment mechanism, which harmonizes task-specific latent spaces while preserving discriminative information. This coordinated design allows the model to learn physiologically consistent representations that remain robust under motion artifacts and signal degradation.

Rather than positioning PPG as a diagnostic substitute for ECG, the proposed framework is intended to enhance signal reliability in wearable environments, enabling PPG to function as a practical complementary modality for AF screening, while ECG remains necessary for definitive clinical diagnosis.

## Materials and methods

### Clinical background: types of atrial fibrillation (AF)

Atrial fibrillation (AF) is commonly categorized into paroxysmal AF (self-terminating episodes, typically < 7 days), persistent AF (lasting > 7 days or requiring cardioversion), long-standing persistent AF (continuous AF for > 12 months), and permanent AF (accepted AF with no further rhythm-control attempts). In practice, PPG-based AF algorithms are primarily used for screening and rhythm surveillance, and positive findings are usually confirmed via ECG-based evaluation. In the datasets used in this study, AF is provided as a rhythm label without clinical subtype annotations; therefore, our objective is binary AF screening (AF vs. non-AF) rather than AF subtype classification.

### Data sources and preprocessing

Two publicly available photoplethysmography (PPG) datasets were used to develop and evaluate the proposed framework: an internal dataset for model training/validation and an external dataset for cross-domain verification.

The internal dataset was derived from Liu et al. [26] and comprises 30,773 nonoverlapping 10-s PPG segments collected from 91 patients who underwent radiofrequency ablation. The signals were recorded at 100 Hz and annotated into six rhythm types: sinus rhythm (SR), premature ventricular contraction (PVC), premature atrial contraction (PAC), ventricular tachycardia (VT), supraventricular tachycardia (SVT), and atrial fibrillation (AF). For this study, only the SR and AF samples were retained to construct a clinically relevant binary classification task.

The demographic and recording characteristics of the internal cohort are summarized in Table 1. The available variables included age (51.2 ± 12.7 years), sex (male 45/91, 49.5%), BMI (25.4 ± 3.6 kg/m²), systolic/diastolic blood pressure (130.3 ± 14.1/81.9 ± 8.7 mmHg), CHA₂DS₂-VASc score (1.0 [0.0–2.0]), hypertension incidence (18/91, 19.8%), mean recording duration (82.99 ± 65.20 min), and heart rate statistics (median 89.53 ± 21.82 bpm; range 49.94–170.01 bpm). Clinical AF subtype annotations (paroxysmal/persistent/long-standing persistent/permanent) were not provided in the released labels; therefore, the present work focuses on binary AF screening rather than subtype classification.

To ensure subject-level independence and prevent data leakage, patients were split into training, validation, and testing sets. The distributions of the SR and AF segment counts across splits are summarized in Table 2 (patient-level division).

An external test set was extracted from the MIMIC-III waveform database to evaluate generalizability under domain shift. It contained PPG recordings from 35 ICU patients (19 AF patients and 16 non-AF patients) sampled at 125 Hz (mostly finger PPG recorded by bedside monitors). Each recording was segmented into nonoverlapping 10-s windows. No subjects overlapped between the internal and external datasets, ensuring independent domain distributions. Patient-level demographics, comorbidities, medication records, and clinical AF subtype annotations are not available in the utilized waveform subset; therefore, only the available acquisition characteristics and label compositions are summarized in Table 1, whereas dataset split statistics for the internal cohort are reported in Table 2.

Although detailed patient-level demographics and clinical covariates are unavailable in the utilized MIMIC-III waveform subset, this external dataset was intentionally selected to test cross-domain generalizability rather than demographic equivalence. Specifically, our external validation aims to assess whether the proposed framework preserves PPG reconstruction quality and AF screening performance under substantially different acquisition environments (ICU bedside monitoring), sampling protocols (125 Hz), and population characteristics, which better reflect real-world deployment scenarios where PPG data may be collected under heterogeneous conditions with incomplete metadata. Consequently, the absence of demographic comparability does not invalidate the external evaluation; instead, it strengthens the robustness assessment against distribution shifts. The lack of external demographic and subtype information is acknowledged as a limitation and is discussed further in Sect. Data sources and preprocessing

To approximate wearable monitoring conditions in which motion and contact artifacts frequently contaminate PPG signals, synthetic noise was incorporated following Paliakaitė et al. [11]. Five artifact types—device displacement, forearm motion, hand motion, poor contact, and additive Gaussian noise—were applied under controlled signal‒to‒noise ratios (0–20 dB) and durations (4–10 s). This procedure standardizes degradation patterns for robust feature learning rather than serving as an experimental manipulation.

All the signals were subjected to a unified preprocessing pipeline. A fifth-order Butterworth low-pass filter was used to suppress high-frequency noise, and the recordings were resampled to 100 Hz for temporal consistency. Each segment was normalized via Z score transformation on the basis of its own mean and standard deviation to ensure comparable amplitude scales across subjects. Segmentation, normalization, and data splits were performed at the patient level to guarantee statistical independence among the training, validation, and testing sets.

**Table 1:** Demographic, recording, and AF case details of the internal cohort and the external MIMIC-III waveform subset

Variable	Internal cohort (*N* = 91)	External cohort: MIMIC-III subset (*N* = 35)
Age, years	51.2 ± 12.7	Not available
Male, n (%)	45 (49.5)	Not available
BMI, kg/m²	25.4 ± 3.6	Not available
SBP, mmHg	130.3 ± 14.1	Not available
DBP, mmHg	81.9 ± 8.7	Not available
CHA₂DS₂-VASc score	1.0 (0.0–2.0)	Not available
Hypertension, n (%)	18 (19.8)	Not available
Recording duration, minutes	82.99 ± 65.20	Not available
Heart rate (median), bpm	89.53 ± 21.82	Not available
Heart rate range, bpm	49.94–170.01	Not available
AF cases (patient-level), n	70	19
Non-AF cases (patient-level), n	21	16
AF subtype (paroxysmal/persistent/…), n	Not available	Not available
Samples per AF case (segments/patient)	211 [119–324] (range 24–463)	Not available
Sampling rate	100 Hz	125 Hz
Segment length	10 s	10 s
Acquisition setting	Radiofrequency ablation cohort	ICU bedside monitor (mostly finger PPG)

**Table 2:** Distribution of the SR and AF segments across dataset splits (patient-level division)

Subset	Patients	SR samples	AF samples
Training	63	10,510	11,679
Validation	13	1,812	1,959
Testing	15	2,282	2,531

### Model architecture

The proposed framework adopts a dual-task transformer–variational autoencoder (transformer–VAE) architecture to jointly reconstruct photoplethysmography (PPG) waveforms and classify atrial fibrillation (AF) under varying noise and motion artifacts. The model integrates a shared encoder for latent feature extraction and two task-specific decoders: one for signal reconstruction and another for rhythm classification. This design enables end-to-end learning that captures long-range temporal dependencies, enhances noise robustness, and ensures consistency between generative and discriminative representations.

The encoder first processes input PPG sequences through convolutional front-end and adaptive average pooling for local feature extraction and dimensionality reduction. The reduced sequence is then passed into a two-layer transformer encoder comprising multihead self-attention (MHSA) and feedforward network (FFN) blocks, which effectively model nonstationary temporal dynamics such as the irregular beat intervals characteristic of AF. Residual connections and layer normalization are incorporated to stabilize training and maintain gradient flow.

The encoded representations are projected through a fully connected layer to obtain a 64-dimensional shared latent vector, which is subsequently decomposed into two components: a 24-dimensional reconstruction latent vector (*z*_*rec*_) and an 8-dimensional classification latent vector (*z*_*cls*_). The reconstruction branch follows the standard VAE formulation, where *z*_*rec*_ is sampled via the reparameterization trick from a learned Gaussian distribution with mean and log-variance parameters. A Kullback–Leibler (KL) divergence term regularizes this latent space to preserve smoothness and continuity.

To harmonize the two latent subspaces, a projection head maps *z*_*rec*_ and *z*_*cls*_ into a shared 16-dimensional alignment space. A cosine similarity loss is applied between the projected vectors to encourage latent alignment and mitigate task interference. This alignment regularization acts as a controllable coupling mechanism, promoting semantic coherence while allowing each branch to specialize.

The reconstruction decoder comprises transposed convolutional layers that regenerate the clean PPG waveform from *z*_*rec*_, thereby embedding physiological priors into the learning process. The classification decoder is a three-layer fully connected network that maps *z*_*cls*_ to the probability of AF versus sinus rhythm. Together, these branches integrate waveform fidelity and diagnostic precision within a unified representation-learning framework.

The overall objective function combines four terms: reconstruction loss (*L*_*rec*_), classification loss (*L*_*cls*_), KL divergence (*L*_*KL*_), and alignment loss (*L*_*align*_). The total loss is expressed as:

1 $$\:{\mathcal{L}}_{total}=\beta\:\cdot\:{\mathcal{L}}_{rec}+{\mathcal{L}}_{cls}+{\mathcal{L}}_{KL}+\gamma\:\cdot\:{\mathcal{L}}_{align}$$

where *β* and *γ* control the relative importance of the reconstruction and alignment components. This formulation balances task cooperation and specialization, enabling the model to learn generalizable, noise-resilient, and physiologically interpretable features for reliable AF detection.

### Training strategy

Model optimization follows a minibatch stochastic gradient descent scheme using the AdamW optimizer, which is selected for its stable convergence in multitask objectives that combine generative and discriminative learning. Early stopping is applied by monitoring validation reconstruction loss to prevent overfitting and to maintain generalization stability. Mini-batches are uniformly sampled across varying noise types and signal-to-noise ratio (SNR) levels, ensuring exposure to representative signal degradation conditions.

To enhance robustness, stochastic augmentations are incorporated during training, including random amplitude scaling, temporal jittering, and randomized noise placement within each segment. These perturbations introduce controlled variability, guiding the model toward learning invariant and noise-tolerant representations. Batch composition dynamically alternates between clean and corrupted inputs, further promoting adaptation to heterogeneous signal quality without overreliance on a single noise profile.

The total objective integrates four components—reconstruction, classification, Kullback–Leibler (KL) divergence, and alignment losses—with weighting coefficients *β* and *γ* controlling the trade-off between reconstruction fidelity and latent alignment. Binary cross-entropy is adopted for classification, the mean squared error (MSE) is used for reconstruction, the KL divergence between the learned posterior and unit Gaussian priors is used for regularization, and the cosine distance for alignment consistency between latent projections is used.

Training stability is assessed through validation metrics, including the AUC, reconstruction RMSE, and KL divergence. All runs are initialized with multiple random seeds, and averaged results are reported to ensure that the observed trends reflect model robustness rather than initialization bias or sampling variance.

### Structural validation, component analysis, and A-Test validation

To examine the functional contributions of individual architectural components and verify the effectiveness of the multitask design, a set of controlled structural variants was analyzed under identical data, training, and optimization settings. This approach isolates the influence of each module, ensuring that the observed performance differences stem from the architectural configuration rather than from external factors.

Five variants were constructed by selectively removing or modifying key modules within the proposed framework. The classifier-only model omits the reconstruction branch, retaining only the discriminative pathway. The reconstruction-only model excludes the classification branch to isolate the generative capacity of the variational autoencoder. The no-projection variant removes the projection heads, eliminating latent-space mapping between the reconstruction and classification subspaces. The no-alignment model preserves the projection structure but omits the cosine similarity constraint, removing explicit cross-task regularization. Finally, the tiny model reduces parameter capacity while maintaining the full architecture, enabling differentiation between performance gains attributable to structure versus scale.

Each variant was evaluated via consistent reconstruction metrics (RMSE, PSNR, and KL divergence) and classification metrics (AUC, accuracy, precision, sensitivity, and specificity). Among these indicators, the AUC is regarded as the primary diagnostic indicator, while reconstruction quality and heart rate variability (HRV) preservation were examined to assess the physiological fidelity of the generated signals.

In addition to component-level structural validation, we adopted an A-Test validation strategy to evaluate learning stability and structural risk under varying data partition structures. The A-Test examines how classification performance changes when balanced k-fold validation is repeated across multiple fold settings. Rather than relying on a single split, this procedure quantifies the sensitivity of model error to different partition granularities and therefore provides a complementary view of generalization consistency.

This type of repeated partition-based stability analysis is related to robustness-oriented validation strategies discussed in time-series deep learning frameworks [27]. Following this rationale, we use the term “A-Test” in this study to denote a repeated balanced k-fold evaluation procedure for assessing error stability across data splits.

Specifically, we performed repeated balanced k-fold validation under multiple fold configurations, ensuring class balance in each partition. For each fold configuration, classification error was recorded and summarized across repetitions. A smaller fluctuation in error indicates stronger structural stability and lower sensitivity to sampling variation, whereas a lower average error reflects greater learning capacity. The results of the A-Test are reported in section. “A-Test validation: structural risk and learning capacity”, including error trends across fold settings and summary stability statistics.

### Implementation and reproducibility

All the implementations were developed in Python via the PyTorch framework with GPU acceleration. The training pipeline adopted mixed-precision computations to improve efficiency and numerical stability. Signal preprocessing, data augmentation, and visualization were performed via standard scientific libraries, including NumPy, Pandas, and Matplotlib. The motion-artifact generation module was implemented as a configurable wrapper supporting randomized injection of multiple noise patterns and variable signal-to-noise ratio (SNR) levels. Synthetic artifacts were generated on the fly during training to prevent data leakage and maintain variability.

To ensure full reproducibility, all model architectures, preprocessing routines, and training scripts were version-controlled via Git. The hyperparameters, training logs, and evaluation metrics were systematically recorded for every training run. Random seeds for initialization, data shuffling, and augmentation were fixed unless otherwise specified.

The complete codebase—including data loaders, model checkpoints, and artifact templates—can be made available upon reasonable request. A public release with documentation and pretrained models is planned to facilitate future benchmarking and collaborative research on robust PPG-based AF detection under noisy conditions.

## Results

### PPG reconstruction performance

The proposed transformer–VAE framework was first evaluated for its ability to reconstruct clean photoplethysmography (PPG) signals from noise-contaminated inputs. Three competitive baselines—VanillaVAE, UNet1D, and CycleGAN—were included for comparison under both sinus rhythm (Non-AF) and atrial fibrillation (AF) conditions. The quantitative evaluation considered waveform fidelity, heart rate (HR) estimation, and heart rate variability (HRV) preservation. The metrics included the root mean square error (RMSE), peak signal-to-noise ratio (PSNR), HR error, and two time-domain HRV indices—SDNN and RMSSD.

**Fig. 1:**
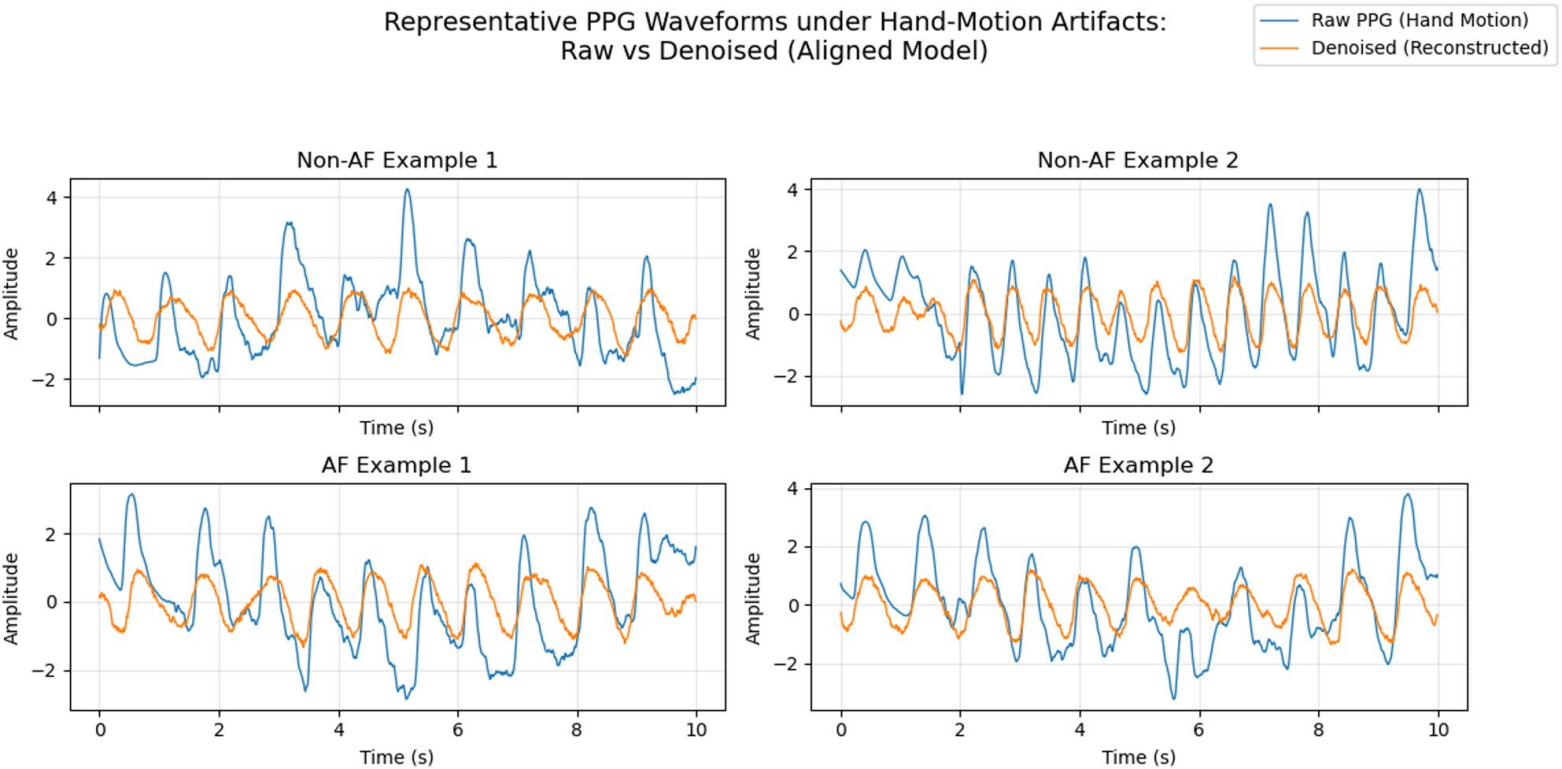
Representative raw and denoised PPG waveforms in the non-AF and AF segments under motion artifacts. Representative 10-s photoplethysmography (PPG) segments affected by motion artifacts are shown for both Non-AF (top row) and AF (bottom row) rhythms. In each panel, the motion-corrupted raw signal (blue) is overlaid with the denoised/reconstructed waveform generated by the proposed model (orange) and aligned on the same time axis for direct comparison. The reconstructed signals exhibit reduced baseline fluctuations and motion-induced distortions while preserving the underlying pulsatile morphology, supporting subsequent quantitative reconstruction analyses

To provide an intuitive visualization of the denoising performance, representative motion-corrupted PPG segments and their reconstructed counterparts are illustrated in Fig. 1 for both rhythm classes.

Before reporting agreement statistics such as Bland–Altman analysis, we quantified the linear association between the reference ECG-derived HR and the HR estimated from the PPG signals before and after denoising within each rhythm class. Table 3 summarizes the Pearson correlation coefficients between clean/reference HRs and noisy and reconstructed PPG-derived HRs. Noisy signals show weak or even negative correlations with the ECG reference (Non-AF: *r* = 0.1228; AF: *r* = − 0.1318), indicating severe degradation in HR estimation under motion artifacts. In contrast, denoising substantially improved the correlation in both rhythm classes (Non-AF: *r* = 0.7978; AF: *r* = 0.7189), demonstrating recovery of physiologically meaningful HR information.

**Table 3:** Pearson correlation between clean reference and noisy/reconstructed HR measurements stratified by rhythm class

Rhythm class	*r* (clean vs. noisy)	*r* (clean vs. reconstructed)	Δr
Non-AF	0.1228	0.7978	0.6750
AF	-0.1318	0.7189	0.8507

Pearson correlation coefficients (r) were used to quantify the associations between the clean/reference heart rate (HR) and the HR derived from motion-corrupted noisy PPG or denoised/reconstructed PPG within each rhythm class. Δr represents the improvement in correlation after denoising (r_reconstructed − r_noisy). A positive Δr indicates enhanced agreement with the clean reference following signal reconstruction

**Fig. 2:**
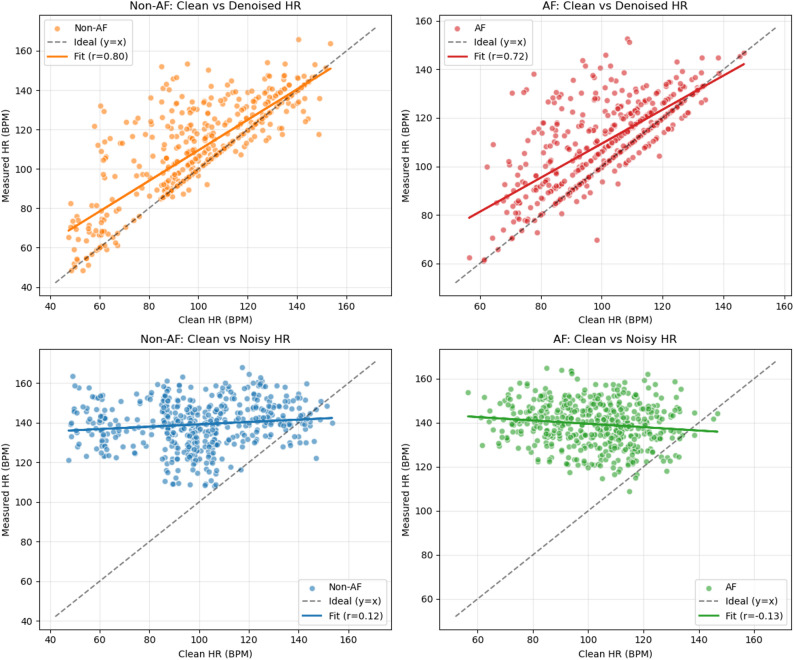
Regression analysis of clean versus noisy and reconstructed HR measurements stratified by rhythm class. Scatter plots illustrate the relationship between the clean/reference heart rate (HR) (x-axis) and the HR derived from the reconstructed PPG (top row) or motion-corrupted noisy PPG (bottom row) (y-axis), separately for the Non-AF (left column) and AF (right column) segments. The dashed line represents the ideal identity relationship (y = x), whereas the solid line indicates the fitted regression model. Compared with noisy signals, denoised/reconstructed signals demonstrate a markedly stronger linear association with the clean reference, indicating improved HR consistency across rhythm classes

To further visualize how denoising strengthens the agreement with the reference signal, regression analysis comparing clean/reference HR with noisy and reconstructed PPG-derived HR is presented in Fig. 2. The regression plots illustrate markedly tighter clustering around the identity relationship (y = x) after reconstruction, indicating a stronger linear association and improved physiological consistency across both the Non-AF and AF segments.

As shown in Fig. 2, reconstructed HR measurements align more closely with the identity relationship (y = x) and exhibit stronger linear consistency with the ECG reference than noisy HR measurements do for both Non-AF and AF segments.

**Fig. 3:**
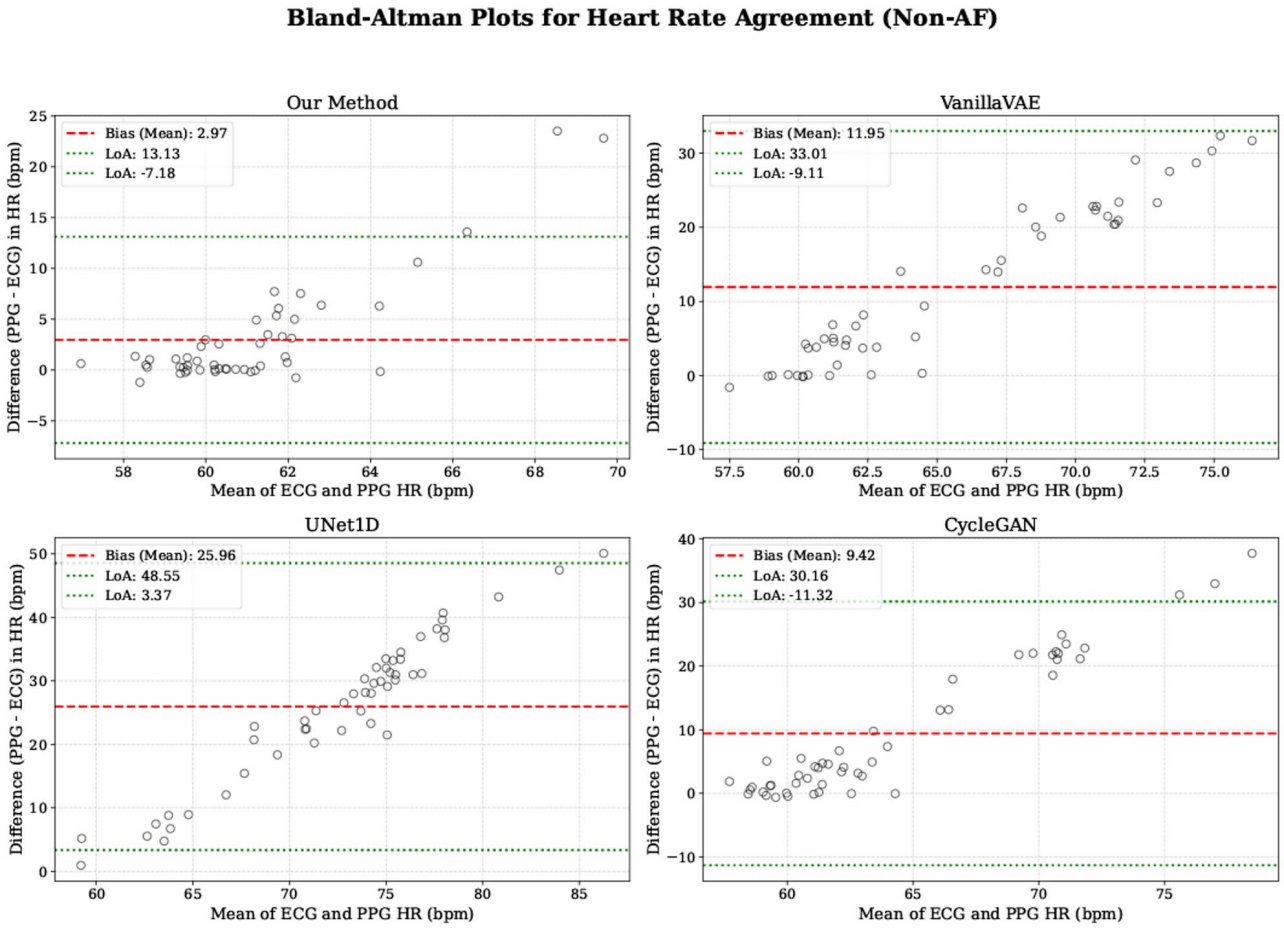
Bland–Altman plots of HR estimation under non-AF conditions. Our method (blue) shows minimal bias and the narrowest 95% limits of agreement, indicating high accuracy and consistency compared with the baselines

Agreement between the reconstructed PPG-derived HR and reference ECG HR was then evaluated via Bland–Altman analysis, with representative plots shown in Fig. 3 for sinus rhythm (non-AF) segments.

Under sinus rhythm conditions, the proposed model achieved the most balanced reconstruction performance across waveform fidelity and physiological metrics. As summarized in Table 4, although VanillaVAE reported a marginally lower RMSE (0.5444 vs. 0.5552), our method substantially improved the HR estimation accuracy (3.10 bpm vs. 12.03 bpm for VanillaVAE and 25.96 bpm for UNet1D) and HRV preservation (SDNN error: 66.15 ms; RMSSD error: 104.78 ms). These improvements indicate superior retention of beat-to-beat variability and physiological dynamics.

Consistent with these quantitative findings, the Bland–Altman analysis in Fig. 3 demonstrated minimal bias (2.97 bpm) and narrow limits of agreement (− 7.18 to 13.13 bpm), confirming stable HR reconstruction relative to the ECG reference.

**Table 4:** Performance comparison of the reconstructed PPG signals under non-AF conditions

Model	RMSE (Mean ± Std)	PSNR (dB)	HR Error (bpm)	SDNN Error (ms)	RMSSD Error (ms)
Our Method	0.5552 ± 0.0588	11.70 ± 0.92	3.10 ± 5.06	66.15 ± 53.30	104.78 ± 75.08
VanillaVAE	0.5444 ± 0.0755	11.90 ± 1.27	12.03 ± 10.55	143.67 ± 79.76	201.14 ± 102.81
UNet1D	0.7236 ± 0.0467	9.37 ± 0.78	25.96 ± 11.41	212.36 ± 39.69	269.42 ± 67.40
CycleGAN	0.5648 ± 0.0571	11.55 ± 1.01	9.49 ± 10.41	124.31 ± 75.76	174.21 ± 93.98

RMSE: root mean square error; PSNR: peak signal-to-noise ratio; HR: heart rate; SDNN: standard deviation of NN intervals; RMSSD: root mean square of successive differences. The values are reported as the means ± standard deviations

Our model demonstrates minimal bias (2.97 bpm) and narrow limits of agreement (− 7.18 to 13.13 bpm), indicating stable HR reconstruction.

**Fig. 4:**
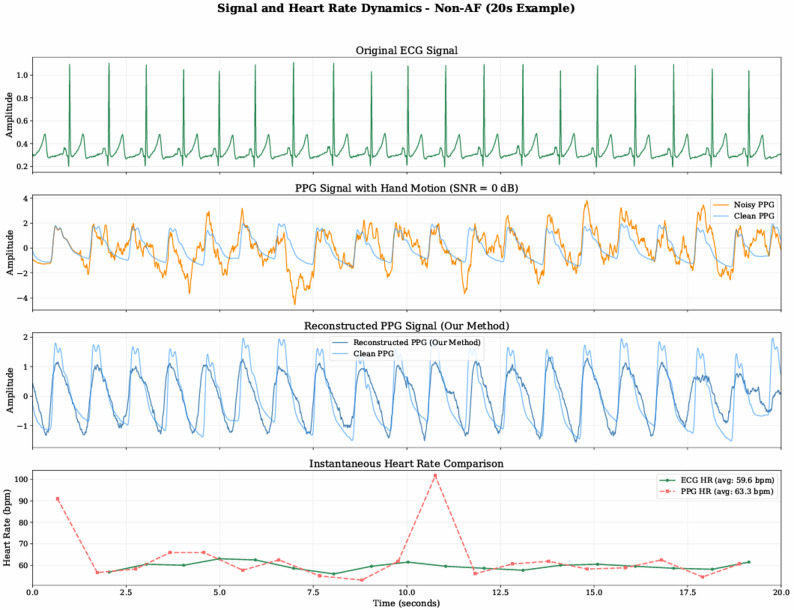
Example of reconstructed PPG waveforms under non-AF conditions. Our model preserves waveform details and HR fluctuations closely matching the clean reference, whereas baseline models show visible smoothing artifacts

A visual waveform comparison further supports these findings, as illustrated in Fig. 4, where the reconstructed non-AF PPG signals generated by the proposed model closely preserve the waveform morphology and heart rate fluctuations relative to those of the clean reference, whereas the baseline methods exhibit visible smoothing artifacts.

The reconstructed signals closely follow physiologic pulsatile patterns, whereas the baseline models exhibit smoothing artifacts that distort HR dynamics.

AF reconstruction presents additional challenges owing to intrinsic rhythm irregularity. While UNet1D and CycleGAN achieve slightly lower mean HR errors (~ 4.5 bpm), their reconstructions oversmooth beat-to-beat variability, resulting in substantially degraded HRV fidelity. As summarized in Table 5, the proposed model better preserves the temporal irregularity characteristic of AF, achieving the lowest SDNN (30.55 ms) and RMSSD (46.47 ms) errors. These findings indicate superior retention of physiologically meaningful beat-to-beat variability under arrhythmic conditions.

**Fig. 5:**
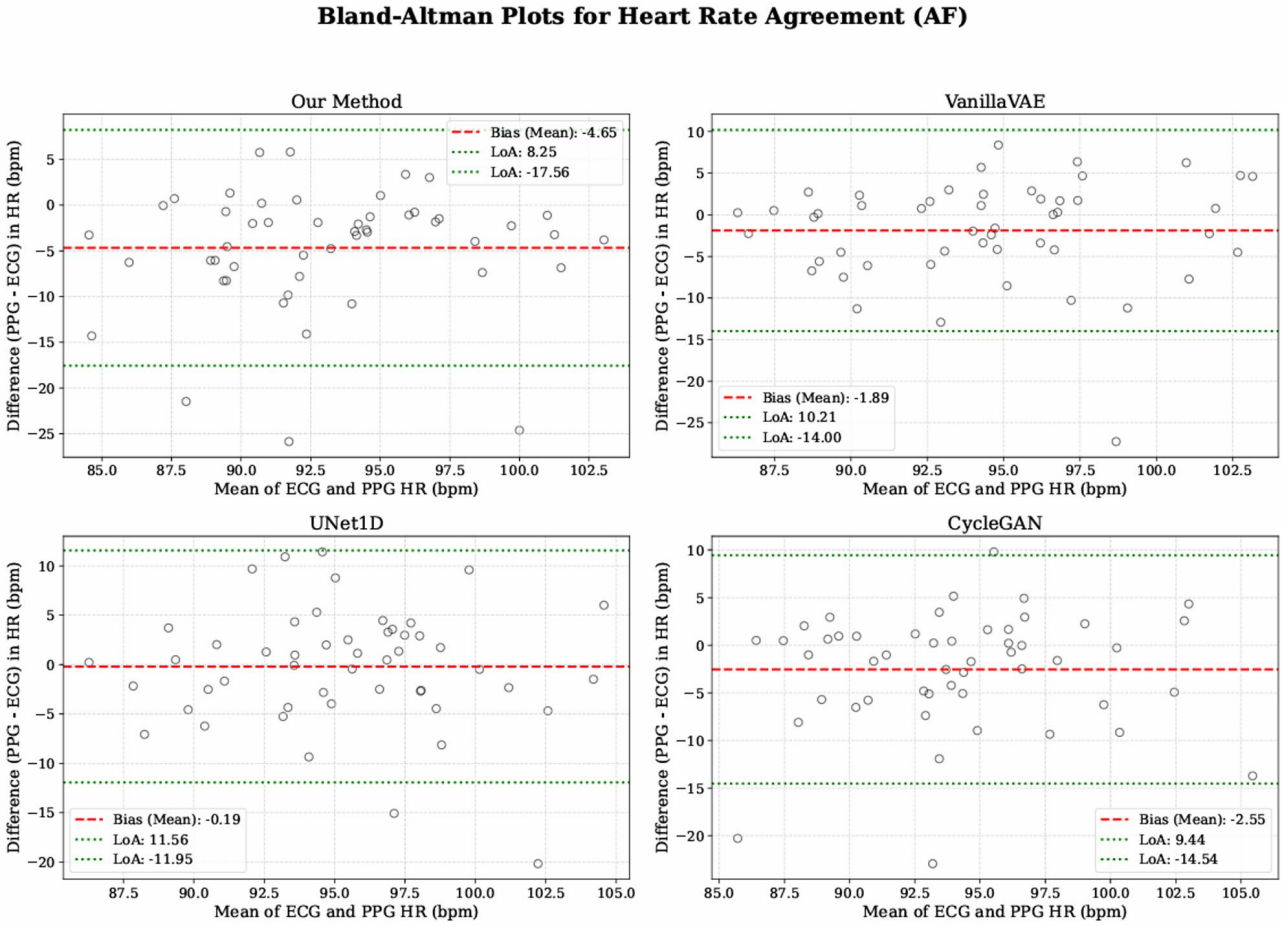
Bland–Altman plots of HR estimation under AF conditions. Despite inherent rhythm irregularity in AF, our model retains robust agreement with ECG-derived HR, with moderate bias and reduced variance

Bland–Altman analysis further confirmed the robust HR agreement between the reconstructed PPG-derived measurements and the ECG reference under AF conditions, as illustrated in Fig. 5. Despite intrinsic rhythm irregularity, the proposed model maintains consistent agreement with limited bias and controlled variability relative to baseline methods

**Table 5:** Performance comparison of the reconstructed PPG signals under AF conditions

Model	RMSE (Mean ± Std)	PSNR (dB)	HR Error (bpm)	SDNN Error (ms)	RMSSD Error (ms)
Our Method	0.6630 ± 0.1129	11.18 ± 1.73	5.53 ± 5.79	30.55 ± 30.34	46.47 ± 41.83
VanillaVAE	0.5848 ± 0.1127	12.30 ± 1.86	4.53 ± 4.52	42.99 ± 44.84	68.96 ± 59.29
UNet1D	0.7150 ± 0.0532	10.43 ± 1.27	4.41 ± 3.98	44.38 ± 41.20	70.32 ± 60.60
CycleGAN	0.5709 ± 0.0872	12.45 ± 1.41	4.51 ± 4.78	35.05 ± 37.49	56.87 ± 53.59

Bias remains limited (− 4.65 bpm) with tight agreement intervals (− 17.56 to 8.25 bpm), demonstrating resilience to AF-induced variability.

**Fig. 6:**
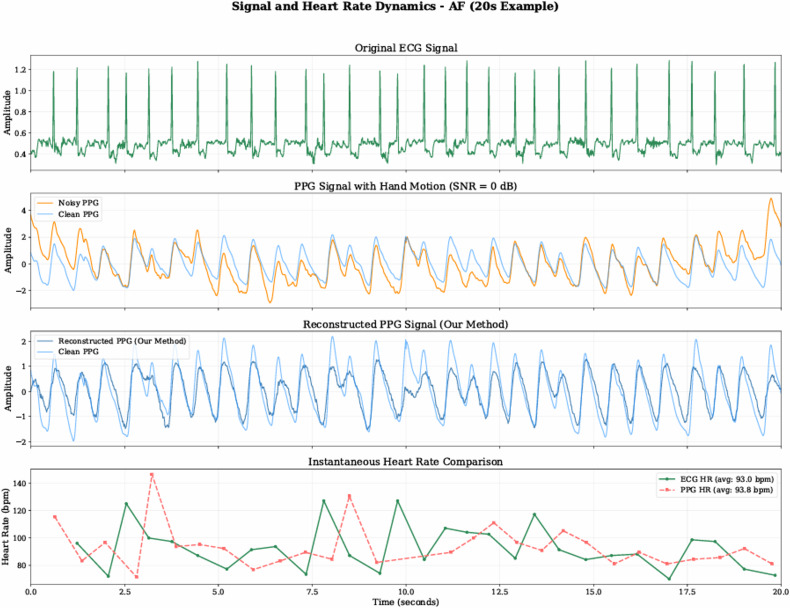
Example of reconstructed PPG waveforms under AF conditions. Only our model replicates the erratic temporal features of AF; other models fail to capture these variations

Waveform-level inspection further highlights physiologic realism under arrhythmic conditions, as illustrated in Fig. 6. The reconstructed AF signals generated by the proposed model preserve irregular beat-to-beat timing and morphology consistent with AF physiology, whereas baseline reconstructions oversmooth temporal variations and fail to capture clinically relevant rhythm irregularities.

### Classification performance

The classification capability of the proposed aligned model was evaluated against CNN1D, ResNet1D, and LSTM_CNN via identical data partitions and preprocessing. The metrics included accuracy, AUC, precision, recall, F1 score, sensitivity, specificity, positive predictive value (PPV), and negative predictive value (NPV) (Table 6).

**Table 6:** Classification performance comparison across different models on the internal dataset

Model	Accuracy	AUC	Precision	Recall	F1-Score	Sensitivity	Specificity	PPV	NPV
Aligned	0.9863	0.999	0.9984	0.9755	0.9868	0.9755	0.9982	0.9984	0.9735
CNN1D	0.9821	0.9988	0.9766	0.9897	0.9831	0.9897	0.9737	0.9766	0.9884
ResNet1D	0.9938	0.9997	0.9976	0.9905	0.9941	0.9905	0.9974	0.9976	0.9896
LSTM_CNN	0.9884	0.9987	0.9866	0.9913	0.989	0.9913	0.9851	0.9866	0.9903

AUC = area under the curve; PPV = positive predictive value; NPV = negative predictive value

The aligned model achieved leading or comparable scores across all the metrics, particularly for false-positive control (precision = 0.9984, specificity = 0.9982, PPV = 0.9984). These outcomes are clinically meaningful for AF screening, where false alarms may lead to unnecessary interventions. Although ResNet-1D obtained slightly higher AUC (0.9997 vs. 0.9990) and accuracy (0.9938 vs. 0.9863) values, the designed model delivered a more balanced trade-off between precision and recall (F1 = 0.9868, recall = 0.9755).

**Fig. 7:**
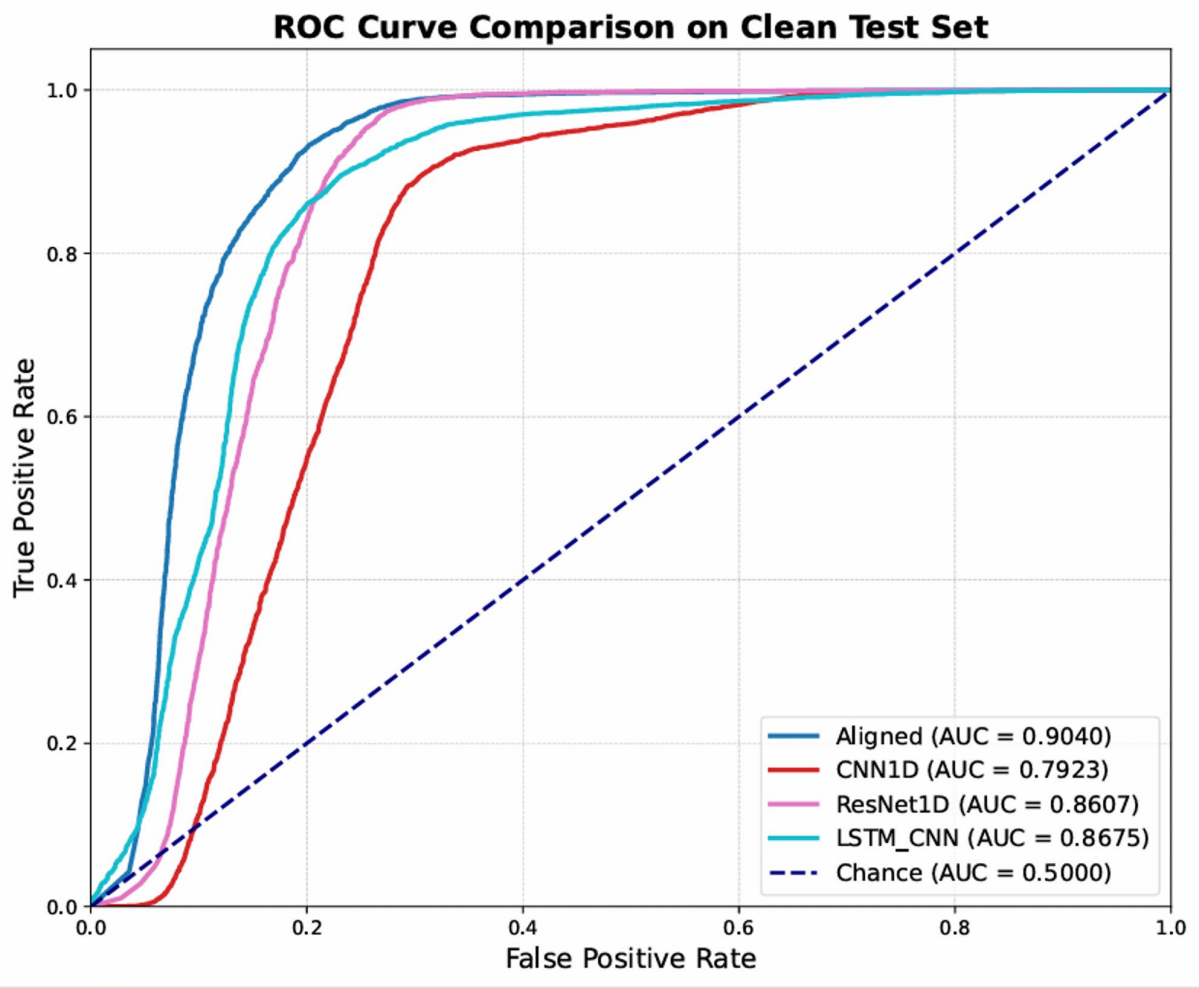
Receiver operating characteristic (ROC) curve comparison of different models on the internal clean test set. The ROC curves illustrate the classification performance of four models—Aligned, CNN1D, ResNet1D, and LSTM_CNN—on the internal test set. The aligned model consistently achieves the highest true positive rate across all false positive rates, with an AUC of 0.9040, surpassing all baselines. CNN1D shows the weakest discriminative ability (AUC = 0.7923), whereas ResNet1D (AUC = 0.8607) and LSTM_CNN (AUC = 0.8675) demonstrate intermediate performance. The diagonal line represents the random classifier baseline (AUC = 0.5)

ROC analysis (Fig. 7) demonstrated that aligned maintains the highest true positive rate across all false positive thresholds. Compared with LSTM_CNN (AUC = 0.8675), aligned improves the AUC by 3.65%, particularly in the low-FPR range critical for screening applications.

To assess domain robustness, all the models were tested on the external MIMIC_Perform_AF dataset containing real-world clinical signals. Aligned maintained the highest overall accuracy (0.8655) and AUC (0.9040), whereas CNN1D underperformed (AUC = 0.7923). These results confirm that the multitask, alignment-regularized architecture generalizes effectively across data distributions and maintains stable diagnostic reliability under real-world variability.

### A-Test validation: structural risk and learning capacity

To further evaluate model stability, structural risk, and learning capacity beyond conventional single-split validation, we applied the A-Test validation framework. The A-Test examines how classification error varies across multiple balanced k-fold partitions, thereby characterizing the sensitivity of a model to sample redistribution. Rather than relying on a single accuracy estimate, this procedure quantifies performance fluctuation as a proxy for structural risk and generalization stability.

Specifically, balanced k-fold cross-validation was repeated across multiple fold configurations (k = 4, 8, 16, 32, 64, 128). For each k, the classification error $\:{E}_{k}$ was computed under identical training conditions. A model exhibiting lower mean error and reduced variability across k values is considered to have lower structural risk and stronger learning capacity.

**Fig. 8:**
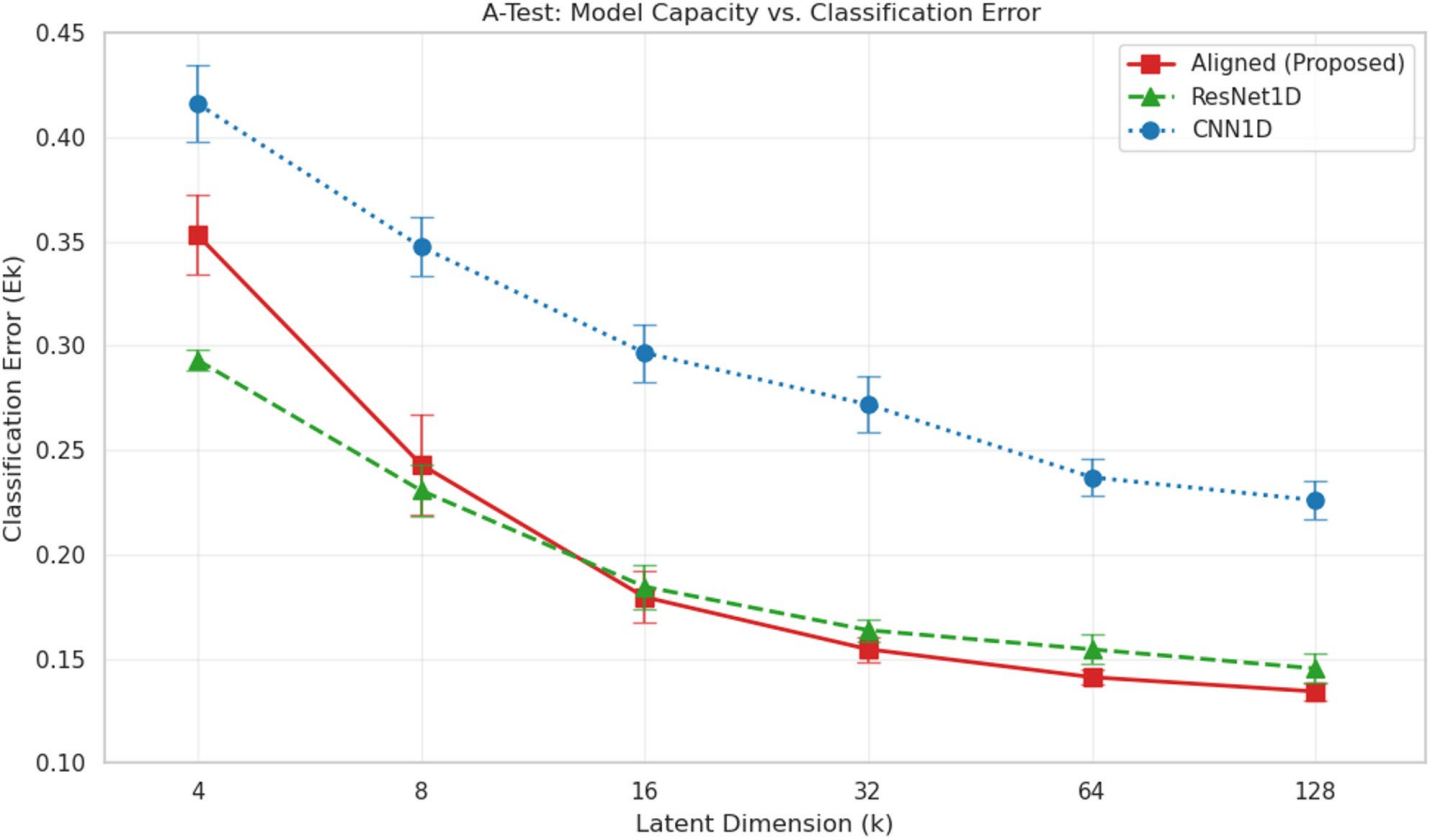
A-Test classification error versus k-fold partition size. The classification error $\:{E}_{k}$ is plotted against the balanced k-fold partitions for the aligned (proposed), ResNet1D, and CNN1D models. The error bars indicate variability across folds. The proposed model demonstrates faster stabilization and reduced fluctuation as k increases, indicating lower structural risk and stronger learning capacity relative to the baselines

Figure 8 illustrates the relationship between model capacity (k) and classification error for the proposed aligned model and two representative baselines (ResNet1D and CNN1D). As k increases, all the models exhibit a decreasing error due to the improved sampling resolution; however, the proposed model consistently maintains a lower error variance and a steeper stabilization trend

The quantitative A-Test summary statistics are presented in Table 7. These metrics include the mean error across k values, minimum and maximum observed errors, standard deviation, absolute performance span (Max–Min), and relative span $\:(\mathrm{Max}-\mathrm{Min})/\mathrm{Max}$. Smaller span values indicate improved structural consistency and robustness to sampling variation.

**Table 7:** A-Test structural risk and learning capacity summary

Model	Mean(Ek)	Min(Ek)	Max(Ek)	SD(Ek)	Max–Min	(Max–Min)/Max
Aligned (Proposed)	0.201	0.1342	0.3533	0.0771	0.2191	0.6201
ResNet1D	0.1953	0.1453	0.2932	0.0518	0.148	0.5046
CNN1D	0.2992	0.226	0.4162	0.0659	0.1903	0.4571

Ek denotes the classification error obtained under balanced k-fold validation. The mean (Ek) represents the average structural risk, the SD (Ek) reflects the variability across folds, and Max–Min quantifies the performance span. The relative span indicates sensitivity to partition variation. Lower values suggest improved generalization stability

The proposed Aligned model achieves competitive mean error while maintaining controlled variability across fold configurations. Although ResNet1D results in a slightly lower average error, the aligned model demonstrates a balanced tradeoff between performance and stability, indicating effective structural regularization. CNN1D has a higher mean error and broader fluctuation, reflecting a weaker learning capacity under sampling perturbations.

Collectively, the A-Test results confirm that the proposed architecture maintains stable classification behavior across varying partition granularities, supporting its robustness and reduced structural risk. These findings complement conventional accuracy metrics by explicitly quantifying learning capacity under distributional variation.

### Robustness evaluation

To simulate wearable sensing conditions, five types of motion artifacts—Gaussian noise, device displacement, forearm motion, hand motion, and poor contact—were introduced at six signal‒to‒noise ratios (SNRs): 10, 9, 8, 6, 4, and 2 dB.

**Fig. 9:**
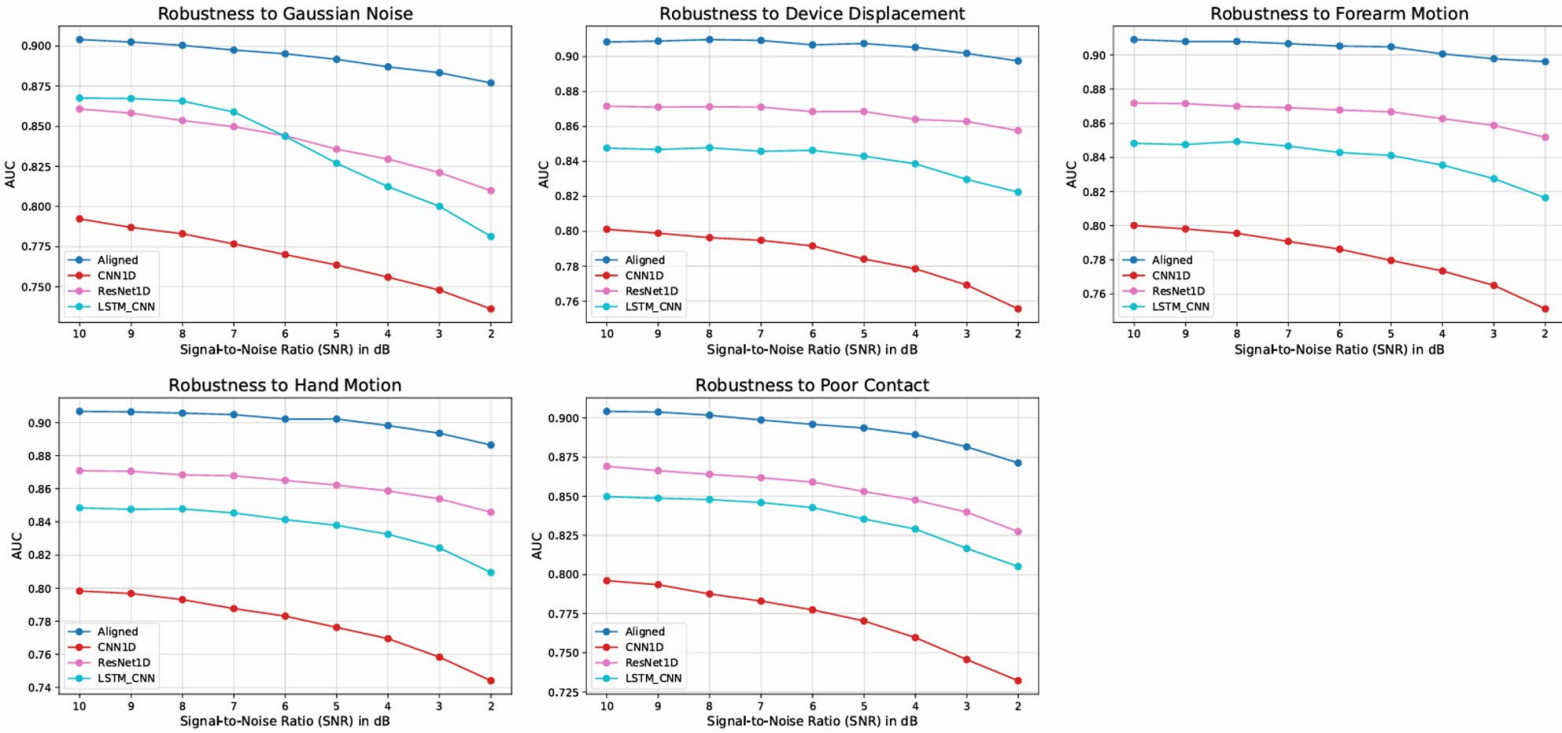
Robustness evaluation of all the models under five types of signal perturbations at varying SNR levels. AUC performance comparison of the aligned, CNN1D, ResNet1D, and LSTM_CNN models under five types of noise artifacts: Gaussian noise, device displacement, forearm motion, hand motion, and poor contact. Each line represents model performance across SNR levels from 10 dB to 2 dB. The aligned model consistently maintains the highest AUC and results in the smallest performance drop, demonstrating superior robustness to signal degradation

Across all the perturbations, the aligned model consistently achieved the highest AUC (Fig. 9). Its performance remained stable even at low SNRs; for example, at 2 dB (poor contact), the AUC decreased to only 0.8713, whereas it was 0.7813 for LSTM_CNN and 0.7321 for CNN1D.

Robustness was quantified via the *Max AUC Drop*, defined as the difference between the clean-test AUC and the lowest noisy-test AUC. As summarized in Table 8, the designed method achieves the smallest degradation (0.0327), substantially outperforming the LSTM_CNN (0.0862). These findings indicate that the model effectively disentangled physiological features from noise, providing strong reliability for ambulatory and wearable AF monitoring.

**Table 8:** Summary of robustness performance under noise-corrupted conditions

Model	AUC (Clean)	Accuracy (Clean)	Avg Noisy AUC	Worst Noisy AUC	Max AUC Drop
Aligned	0.904	0.8655	0.8986	0.8713	0.0327
CNN1D	0.7923	0.78	0.7747	0.7321	0.0602
ResNet1D	0.8607	0.8572	0.8567	0.8099	0.0509
LSTM_CNN	0.8675	0.8258	0.836	0.7813	0.0862

Max AUC Drop = AUC (Clean) − Worst noisy AUC; lower values indicate better robustness

### Ablation study

An ablation analysis of MIMIC_Perform_AF was performed to evaluate the impact of four design factors: feature alignment, projection, model scale, and multitask learning. Five configurations were tested:

- Aligned_all: full model;
- Aligned_tiny: parameter-reduced model;
- No_Projection: without latent projection;
- No_Aligned: without alignment loss;
- Classifier_Only: without a reconstruction branch.

The results (Table 9) highlight the critical role of multitask learning. The *Aligned_all* model achieved an AUC of 0.9097 and an accuracy of 0.8841, whereas *Classifier_Only* decreased to 0.8248 (AUC), confirming a + 10.3% gain from joint reconstruction guidance.

**Table 9:** Ablation study results on the MIMIC_Perform_AF dataset

Model	AUC	Accuracy	Sensitivity	Specificity	Precision
Aligned_all	0.9097	0.8841	0.9477	0.8067	0.8563
Aligned_tiny	0.9225	0.8503	0.9265	0.7577	0.823
NO_Aligned	0.915	0.8532	0.9686	0.713	0.804
NO_Projection	0.9241	0.8491	0.9641	0.7092	0.8013
Classifier_Only	0.8248	0.757	0.7422	0.7749	0.8004

Removing components such as feature alignment or projection slightly improves the AUC but often leads to imbalanced performance (e.g., lower specificity). The fully aligned_all model achieves the most stable and interpretable tradeoff, validating the integrated design

Interestingly, *No_Aligned* yielded a slightly higher AUC (0.9150), suggesting that excessive alignment regularization may constrain representational flexibility. Conversely, *No_Projection* achieved a high AUC (0.9241) but exhibited low specificity (0.7092), implying that projection enhances discriminability by structuring latent subspaces. The compact *Aligned_tiny* model maintained high performance (AUC = 0.9225), validating the scalability of the architecture.

### Joint task coordination analysis

To better understand the coordination dynamics between the reconstruction and classification branches, we conducted a series of targeted experiments and gradient-based analyses. These results reveal how joint training affects performance and where conflicts may arise if task weights are not properly balanced.

We first perform a weight sensitivity experiment on the reconstruction loss weight *α*_*recon*_, and the full results are reported in Table 10. As shown in Table 10, when *α*_*recon*_ = 0, i.e., the reconstruction task is removed, the model exhibits highly imbalanced decision boundaries. The sensitivity increased sharply to 0.9553, but the specificity decreased to 0.1316. This indicates that without reconstruction as a regularizing constraint, the classification branch tends to overfit positive samples, leading to poor generalizability and an extremely skewed decision boundary. On the other hand, when *α*_*recon*_ = 2.0, the reconstruction task becomes overly dominant, causing the classifier to degrade into predicting all samples as positive (sensitivity = 1.0, specificity = 0), and the AUC decreases to 0.5956. To quantify the degree of task conflict, we define the task conflict index (TCI) as:

2 $$\:\mathrm{T}\mathrm{C}\mathrm{I}=\frac{|\mathrm{Sensitivity}-\mathrm{Specificity}|}{\mathrm{Sensitivity}\times\:\mathrm{Specificity}}$$

Under extreme weights (*α*_*recon*_ = 0 and *α*_*recon*_ = 2.0), the TCI reaches 6.54 and ∞, respectively, indicating severe intertask interference. In contrast, within the moderate range of *α*_*recon*_∈[0.3,1.5], the average TCI is only 0.23, suggesting that a balanced weight assignment significantly mitigates conflict between tasks and promotes joint optimization.

After determining the optimal reconstruction weight (*α*_*recon*_ = 0.9), we further examined the effect of the feature alignment loss weight *α*_*align*_. Table 11 shows that model performance exhibits a clear inverse-U shaped curve with respect to *α*_*align*_, reaching its peak at *α*_*align*_ = 0.15. In this configuration, the AUC improves from 0.8522 (no alignment) to 0.8608 (+ 1.01%), the test accuracy increases by 0.33%, and the specificity increases by 0.70% without sacrificing sensitivity, resulting in a more balanced and clinically meaningful classifier.

**Fig. 10:**
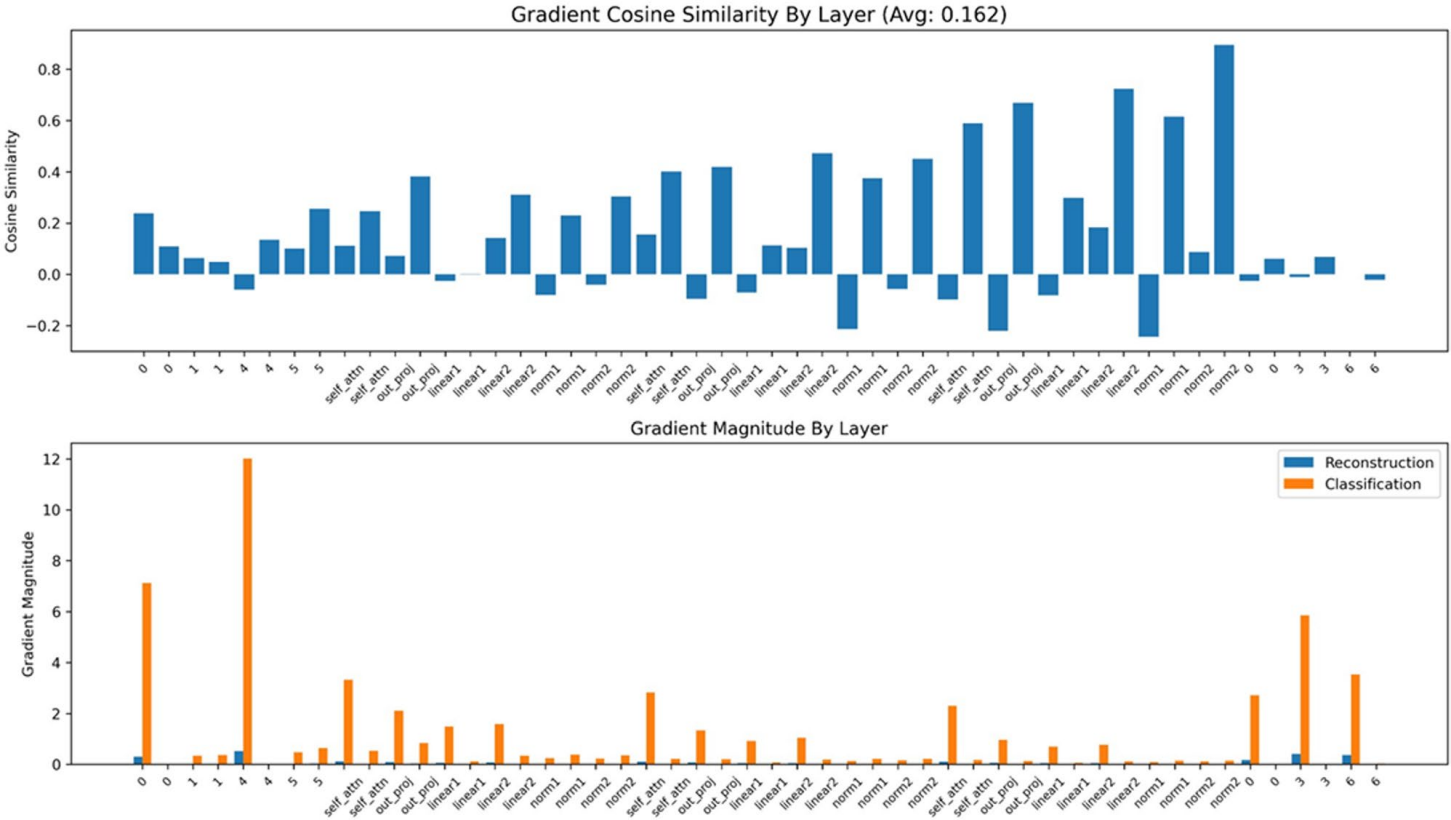
Gradient analysis without feature alignment. Cosine similarity of gradients across encoder layers (*average* = 0.162). Bottom: Gradient magnitude per layer shows the dominance of classification updates

Most notably, gradient-flow analysis (Fig. 10) provides direct evidence that feature alignment is necessary to reconcile training dynamics across the two tasks. As shown in Fig. 10 (top panel), when the alignment loss is disabled, the cosine similarity between the reconstruction and classification gradients across the encoder layers is low, with an average of 0.162, indicating substantial directional disagreement during optimization. Figure 10 (bottom panel) further shows that gradient magnitudes are heavily skewed toward the classification branch across multiple layers, suggesting that classification updates dominate shared representations and can suppress reconstruction-driven regularization.

**Fig. 11:**
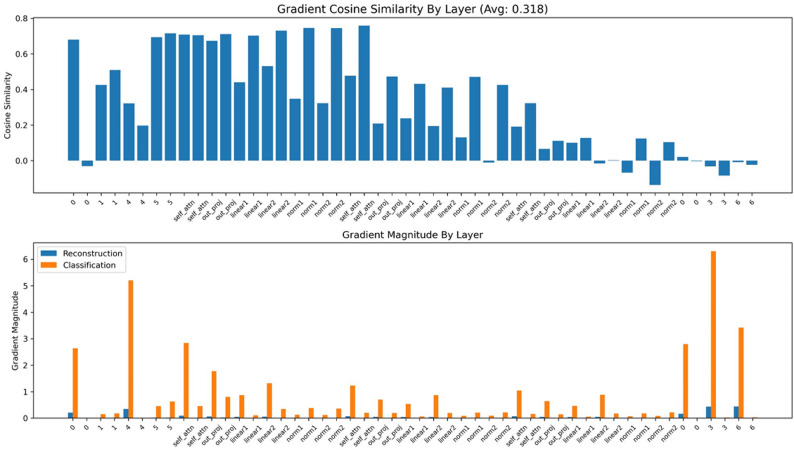
Gradient analysis with feature alignment (*α*_*align*_ = 0.15). The gradient cosine similarity increases to 0.318, indicating improved task coordination. Bottom: Gradient magnitudes become more balanced across tasks

After enabling feature alignment, the gradient directions become noticeably more consistent: the average cosine similarity increases to 0.318 (see Fig. 11), and gradient magnitudes across layers are better balanced—particularly in the shallow encoder layers—indicating improved sharing of representational space between reconstruction and classification. Together, Figs. 10 and 11 confirm that the feature alignment mechanism acts as an effective bridge, reducing intertask interference and enhancing multitask synergy.

**Table 10:** Effect of the reconstruction loss weight (*α*_*recon*_) on task performance and conflict

α_recon_	Val Acc	Test Acc	Test AUC	Test F1	Sensitivity	Specificity	TCI
0	0.8719	0.5728	0.6681	0.7055	0.9553	0.1316	6.54
0.3	0.9756	0.7969	0.8511	0.8171	0.847	0.7392	0.17
0.6	0.9196	0.6588	0.6807	0.702	0.7502	0.5533	0.47
0.9	0.9568	0.8059	0.8522	0.8303	0.8866	0.7127	0.2
1.2	0.9369	0.7957	0.8233	0.8225	0.8838	0.6941	0.27
1.5	0.9711	0.798	0.8706	0.8059	0.8216	0.7492	0.18
2	0.5195	0.5357	0.5956	0.6976	1	0	∞

TCI = |Sensitivity − specificity|/(sensitivity × specificity); ∞ indicates division by zero

**Table 11:** Effect of the feature alignment weight (*α*_*align*_) on model performance (*α*_*recon*_ = 0.9)

α_align_	Val Acc	Test Acc	Test AUC	Sensitivity	Specificity	Test F1
0.0001	0.9767	0.7891	0.8342	0.8542	0.714	0.8127
0.001	0.9798	0.7827	0.847	0.8425	0.7137	0.8059
0.01	0.9775	0.7994	0.8458	0.8786	0.708	0.8243
0.1	0.9788	0.7969	0.8417	0.8464	0.7399	0.817
0.15	0.9801	0.8086	0.8608	0.8873	0.7177	0.8324
0.2	0.9796	0.7901	0.8403	0.8468	0.7247	0.8121

## Discussion

Photoplethysmography (PPG) has become a cornerstone of wearable health monitoring, offering continuous and noninvasive cardiovascular assessment in daily environments. However, its clinical reliability remains limited by motion artifacts, ambient light interference, and perfusion variability, particularly in ambulatory conditions [28–31]. Prior approaches have attempted to mitigate these issues via traditional filtering, ICA-based decomposition [32, 33], or deep learning pipelines such as CNN-RNN hybrids [34, 35]; however, their robustness often degrades under extreme noise and subject variability. As recent work emphasized that motion-induced corruption remains a major obstacle for arrhythmia screening and blood pressure estimation [36–38], there is a critical need for end-to-end architectures that can jointly enhance signal fidelity and diagnostic interpretability. Importantly, while PPG enables continuous and unobtrusive monitoring, electrocardiogram (ECG) remains the clinical gold standard for AF diagnosis, and PPG-based approaches should be interpreted within a complementary screening framework.

The proposed dual-task transformer–VAE framework addresses this challenge by integrating waveform reconstruction and atrial fibrillation (AF) classification within a unified learning paradigm. This bidirectional coupling bridges the long-standing gap between signal recovery and physiological discrimination. The classification objective acts as a physiological regularizer for the reconstruction branch, improving the morphological stability and reducing the average heart rate error to 3.10 bpm—outperforming most prior deep architectures. Conversely, the auxiliary reconstruction task enriches the latent representation with temporal and structural priors, increasing the classification AUC by more than 10% (0.8248 → 0.9097). These findings corroborate prior evidence from multitask learning in biosignal analysis, where shared representations enhance both task-specific performance and domain generalization [5–40]. Importantly, we position these gains as evidence that joint reconstruction–classification learning can improve robustness under controlled degradations rather than as definitive proof of everyday wearable performance across all consumer devices.

Beyond the dual-task formulation, the integration of a gradient-alignment module plays a pivotal role in harmonizing optimization dynamics. Gradient flow analysis revealed a substantial increase in intertask cosine similarity, reflecting improved consistency between the classification and reconstruction gradients. Although the numerical AUC improvement (+ 1.0%) may appear modest, such enhancement is clinically meaningful in safety-critical applications, where even small performance gains can translate into fewer false alarms and missed detections [41, 42]. The alignment constraint thus serves as an implicit coordination mechanism that stabilizes training and reinforces physiologically coherent feature learning. This architectural complexity should be justified over simpler alternatives; therefore, we interpret the alignment module as a targeted mechanism to reduce task interference in noisy PPG, and we explicitly acknowledge that future comparisons against contemporary PPG-AF–specific methods are required to establish the most cost-effective design.

The sensitivity analysis of the loss weights further elucidates the intrinsic trade-offs of multiobjective optimization. Eliminating the reconstruction term (αrecon = 0) yielded nearly perfect sensitivity but poor specificity, demonstrating overfitting to positive cases. Conversely, excessive emphasis on reconstruction (αrecon = 2.0) collapsed discriminative performance. To quantify this imbalance, we introduced the task conflict index (TCI), which reveals extreme instability at both ends of the weighting spectrum but equilibrium within a moderate range (αrecon ≈ 0.9, TCI ≈ 0.23). This observation aligns with theoretical insights from the multitask optimization literature, underscoring the importance of calibrated weight scheduling for mitigating intertask interference and preserving representation diversity [43–45]. We agree that such sensitivity can be a practical drawback for deployment; accordingly, our results suggest a “stable operating zone” (rather than a single optimal point) where performance remains near peak, and future work will prioritize automated or adaptive weighting strategies (e.g., uncertainty-based or gradient-norm balancing) to reduce dataset-specific tuning requirements.

A similar equilibrium emerges in the analysis of the alignment weight (αalign). The performance followed an inverted-U profile, peaking at αalign = 0.15. Moderate alignment promotes consistency between task subspaces, whereas overconstraining latent representations impairs sensitivity and specificity by suppressing task-specific nuances. This mirrors phenomena reported in domain adaptation and adversarial alignment studies, where excessive feature coupling reduces generalizability [46, 47]. Thus, the optimal strategy lies in maintaining a soft alignment that guides but does not dominate task interactions. From a translational perspective, we recommend reporting and validating a small set of default weights and verifying that performance remains stable within a neighborhood, which is a prerequisite for robust use across new devices and populations.

In addition, A-Test validation provides a complementary perspective on model reliability by examining how classification error varies across different balanced k-fold partition settings. The observed error–k trend suggests that the proposed model maintains relatively consistent performance under changing data partitions, indicating lower structural risk and stable learning behavior rather than sensitivity to a specific split. Notably, the remaining fluctuation across k is consistent with the inherent heterogeneity of PPG signals and label noise under artifact corruption; nevertheless, the overall stability pattern supports the claim that dual-task coupling and alignment act as regularizers that mitigate split-dependent variance. At the same time, we interpret the A-Test as an internal stability check rather than a substitute for external, real-world validation of ambulatory wearable recordings.

Comparative evaluation further emphasized the distinctiveness of the proposed joint-task paradigm. Traditional AF detection pipelines typically separate denoising and classification, treating signal restoration as a preprocessing step rather than an integral learning objective [48, 49]. Others rely heavily on handcrafted features or adversarial networks, which either limit scalability or compromise interpretability. In contrast, our model achieves robustness across diverse artifact conditions without clean supervision or explicit segmentation, maintaining rhythm integrity even under severe degradation (e.g., 0–2 dB SNR, hand motion scenarios) [50, 51]. This suggests that the model captures physiologically grounded priors rather than memorizing noise patterns—a critical step toward clinically reliable PPG analysis. Nevertheless, we acknowledge that our baseline comparisons mainly include generic architectures; benchmarking against recent PPG-based AF detection models designed specifically for wearable settings is necessary to conclusively quantify the added value of the proposed approach.

Despite its promising results, several limitations should be acknowledged. First, although subjectwise data partitioning was enforced, the diagnostic generalizability of the classifier across patient populations and acquisition devices was not exhaustively validated. Potential sample imbalance or site-specific biases may influence performance estimates. Second, while synthetic artifacts approximate real-world disturbances, the dataset lacks multicenter, device-diverse recordings, which may constrain its external validity. Third, several unmodeled factors—such as ambient illumination shifts, contact pressure variations, and low-perfusion states—remain open challenges for future modeling efforts. In particular, our external evaluation is based on an ICU waveform subset, whereas real-world wearable scenarios involve more dynamic conditions; thus, further validation under free-living environments is required.

Regarding the clinical utility of reconstruction, the present study demonstrated that adding the reconstruction branch improved downstream AF classification and rhythm-related metrics (e.g., HR/HRV consistency), but whether clinicians benefit directly from the reconstructed waveform beyond algorithmic performance has yet to be quantified. Future analyses should therefore distinguish between reconstruction as a training regularizer and its value for clinical interpretation.

From an implementation perspective, integration into the healthcare system can follow a screening-and-confirmation workflow rather than replace ECG-based diagnosis. In practice, the model can be deployed as a front-line screening tool using wearable PPG, while positive findings should be confirmed through ECG-based evaluation, consistent with current clinical practice. This design improves feasibility in routine ambulatory monitoring while maintaining diagnostic reliability.

## Conclusion

This study introduces a transformer–VAE multitask framework that jointly reconstructs photoplethysmography signals and detects atrial fibrillation, improving waveform fidelity and diagnostic robustness under noise-contaminated conditions. By coupling generative reconstruction with discriminative learning and feature alignment, the model achieves strong AF screening performance while preserving physiologically meaningful rhythm characteristics. A-Test validation further indicates stable error behavior across balanced data partitions, supporting the learning consistency of the multitask design.

Rather than replacing ECG-based diagnosis, the proposed framework is intended as a screening tool within a wearable monitoring workflow, where PPG-derived risk indicators can guide timely confirmatory ECG evaluation. Future work will focus on validation with real-world wearable recordings, adaptive multitask optimization, and edge deployment to enhance clinical reliability in ambulatory settings.

## Data Availability

The datasets analyzed during the current study are publicly available: Liu et al. dataset: https://github.com/zdzdliu/PPGArrhythmiaDetection MIMIC-III Waveform Database: https://zenodo.org/records/6973963. All the preprocessing scripts and model codes are available from the corresponding author upon reasonable request.

## Abbreviations

PPG: Photoplethysmography
AF: Atrial Fibrillation
ECG: Electrocardiogram
SR: Sinus Rhythm
PVC: Premature Ventricular Contraction
PAC: Premature Atrial Contraction
VT: Ventricular Tachycardia
SVT: Supraventricular Tachycardia
MTL: Multitask Learning
VAE: Variational Autoencoder
KL: Kullback–Leibler
SNR: Signal-to-Noise Ratio
AUC: Area Under the Curve
RMSE: Root Mean Square Error
PSNR: Peak Signal-to-Noise Ratio
HR: Heart Rate
HRV: Heart Rate Variability
SDNN: Standard Deviation of NN Intervals
RMSSD: Root Mean Square of Successive Differences
CNN: Convolutional Neural Network
RNN: Recurrent Neural Network
LSTM: Long Short-Term Memory
MHSA: Multi-Head Self-Attention
FFN: Feed-Forward Network
PPV: Positive Predictive Value
NPV: Negative Predictive Value
TCI: Task Conflict Index
MSE: Mean Squared Error
ICU: Intensive Care Unit
